# Impact of High Risk Drug Use on Hospitalization and Mortality in Older People with and without Alzheimer’s Disease: A National Population Cohort Study

**DOI:** 10.1371/journal.pone.0083224

**Published:** 2014-01-13

**Authors:** Danijela Gnjidic, Sarah N. Hilmer, Sirpa Hartikainen, Anna-Maija Tolppanen, Heidi Taipale, Marjaana Koponen, J. Simon Bell

**Affiliations:** 1 Faculty of Pharmacy, University of Sydney, Sydney, New South Wales, Australia; 2 Departments of Clinical Pharmacology and Aged Care, and Kolling Institute of Medical Research, Royal North Shore Hospital, Sydney, New South Wales, Australia; 3 Sydney Medical School, University of Sydney, Sydney, New South Wales, Australia; 4 Centre for Education and Research on Ageing and Concord RG Hospital, Sydney, New South Wales, Australia; 5 Kuopio Research Centre of Geriatric Care, School of Pharmacy, University of Eastern Finland, Kuopio, Finland; 6 Department of Neurology, University of Eastern Finland, Kuopio, Finland; 7 Sansom Institute, School of Pharmacy and Medical Sciences, University of South Australia, Adelaide, South Australia, Australia; 8 Centre for Medicine Use and Safety, Faculty of Pharmacy and Pharmaceutical Sciences, Monash University, Melbourne, Victoria, Australia; University of Antwerp, Belgium

## Abstract

**Background:**

Evidence is lacking about outcomes associated with the cumulative use of anticholinergic and sedative drugs in people with Alzheimer’s disease (AD). This retrospective cohort study investigated the relationship between cumulative exposure to anticholinergic and sedative drugs and hospitalization and mortality in people with and without AD in Finland.

**Methods:**

Community-dwelling people aged 65 years and over, with AD on December 31^st^ 2005 (n = 16,603) and individually matched (n = 16,603) comparison persons (age, sex, region of residence) were identified by the Social Insurance Institution of Finland. Drug utilization data were extracted from the Finnish National Prescription Register. Exposure to anticholinergic and sedative drugs was defined using the Drug Burden Index (DBI). Hospitalization and mortality data were extracted from national registers. Cox and zero-inflated negative binomial analyses were used to investigate the relationship between DBI and hospitalization and mortality over a one-year follow-up.

**Results:**

In total, 5.8% of people with AD and 3.7% without AD died during 2006. For every unit increase in DBI, the adjusted hazard ratio for mortality was 1.21 (95% confidence intervals [CI]: 1.09–1.33) among people with AD, and 1.37 (95%CI: 1.20–1.56) among people without AD. Overall, 44.3% of people with AD and 33.4% without AD were hospitalized. When using no DBI exposure as the reference group, the adjusted incidence rate ratio for length of hospital stay among high DBI group (≥1) in people with AD was 1.15 (95%CI: 1.05–1.26) and 1.63 (95%CI: 1.41–1.88) in people without AD.

**Conclusion:**

There is a dose-response relationship between cumulative anticholinergic and sedative drug use and hospitalization and mortality in people with and without AD.

## Introduction

Older people are susceptible to adverse drug events (ADEs) due to multi-morbidity, age-related physiological changes, and multiple drug use [1]. In people with dementia, underlying functional impairment may confer greater susceptibility to ADEs including falls, fractures, and excess sedation [2], [3]. Population-based research suggests that older people continue to take drugs with an unfavorable risk to benefit ratio [4]. Despite guidelines advising against the use of drugs with sedative or anticholinergic properties in people with Alzheimer’s disease (AD), drugs with sedative or anticholinergic properties remain widely used in people with AD. Among people with AD in Europe, 23% used anticholinergic drugs with significant or moderate effects [5]. In people with advanced dementia in institutional care in the USA, 28% used antipsychotics and 54% used antidepressants [6]. Potentially inappropriate drugs, defined using the Beers Criteria, were used by 20% of older adults with dementia living in the community in the USA [7]. In Finland, use of antipsychotics is more prevalent among people with AD compared with age and sex matched people without AD [8].

Exposure to anticholinergic and sedative drug classes has been associated with adverse outcomes in older people [9], [10]. In studies of older people, use of drugs with anticholinergic and sedative effects has been associated with impaired physical function, functional status, balance and mobility [11]–[14]. Moreover, cumulative exposure to central nervous system (CNS) drugs has been associated with incident mobility limitation [15]. In people with dementia, use of psychotropic drugs, many of which have anticholinergic and sedative effects, is very common internationally [16]. There is a large body of research focused on ADEs associated with single classes of drugs with sedative and anticholinergic properties. This includes research about mortality associated with antidepressant, antipsychotic and sedative hypnotic use in older people. However, at present, there is a lack of empirical data about possible negative outcomes associated with the cumulative use of both anticholinergic and sedative drug classes in older people with AD compared to those without AD. The Drug Burden Index (DBI) is a validated pharmacological risk assessment tool that measures cumulative exposure to anticholinergic and sedative drugs incorporating the principles of dose-response and maximal effect [17]. Rather than focus on the risk associated with a specific anticholinergic or sedative drug, the DBI takes into account that older people often use many drugs with anticholinergic and sedative properties. The DBI includes drugs with both central and peripheral anticholinergic side-effects. This is important because even peripheral anticholinergic side-effects (e.g. blurred vision, increased heart rate) can be associated with serious adverse outcomes in older people [18]. Increasing DBI has been associated with functional impairment, hospitalization and frailty in older adults [17], [19]–[22]. People with AD may be particularly susceptible to these outcomes.

The availability of large-scale national data about drug exposure in people diagnosed with AD in Finland provides a near unique opportunity to investigate high risk prescribing in this patient population [23], [24]. The objective of this cohort study was to investigate the association between cumulative anticholinergic and sedative drug exposure, measured using the DBI, and hospitalization and mortality in people with and without AD in Finland. The a priori hypothesis was that compared to non-exposed individuals, exposure to anticholinergic and sedative drugs will be associated with higher rates of hospitalization and mortality in both people with and without AD.

## Methods

### Data Sources

The study linked data from the Finnish National Prescription Register, Special Reimbursement Register and the Finnish Hospital Discharge Register. The Finnish National Prescription and Special Reimbursement Registers are maintained by the Social Insurance Institution (SII) of Finland and include records of reimbursed drugs purchased by 5.4 million Finnish residents living in non-institutional setting [23]. The Special Reimbursement Register includes data about diagnosed chronic diseases. For a diagnosis to be included in the Register, a patient’s disease or condition must meet explicit pre-defined criteria, and written documentary evidence must be provided to the SII by the treating physician. The Finnish Hospital Discharge Register is maintained by the National Institute for Health and Welfare and includes records of all inpatient care episodes in Finland [24]. The registers are updated on a daily basis for all deaths that occur in Finland. This is done via the Finnish National Population Information System, maintained by the Population Register Center of Finland. Data linkage for this study was performed by the National Institute for Health and Welfare using each Finnish resident’s unique social security number. According to Finnish laws, no ethics committee approval was required as only de-identified data were provided to the researchers and the study participants were not contacted.

### Study Sample

Using the Special Reimbursement Register, the SII identified all community-dwelling persons (n = 28,093) with a verified diagnosis of AD in Finland on December 31, 2005. To verify a diagnosis of AD a physician must send a medical statement to the SII. The medical statement must state that the person has (i) symptoms consistent with mild or moderate AD, (ii) experienced a decrease in social capacity over a period of at least three months, (iii) received a CT or MRI scan, (iv) had possible alternative diagnoses excluded, and (v) received confirmation of the diagnosis by a registered neurologist or geriatrician. Each medical statement is then assessed by the SII to ensure that a patient meets the criteria of the Diagnostic and Statistical Manual Version IV (DSM-IV) and NINCDS-ADRDA for AD [25]. The Finnish Current Care guidelines recommend that all persons with mild or moderate AD should have their diagnosis verified with the SII and be prescribed drugs for AD. However, having mild or moderate AD is only a requirement for initial verification of the AD diagnosis. The SII does not withdraw the AD diagnosis or eligibility to receive reimbursed drugs for AD when a person later develops severe AD. This meant our sample included persons with mild, moderate and severe AD. Persons with mixed dementias of the AD/vascular and AD/Lewy body disease were included in our sample.

**Figure 1:**
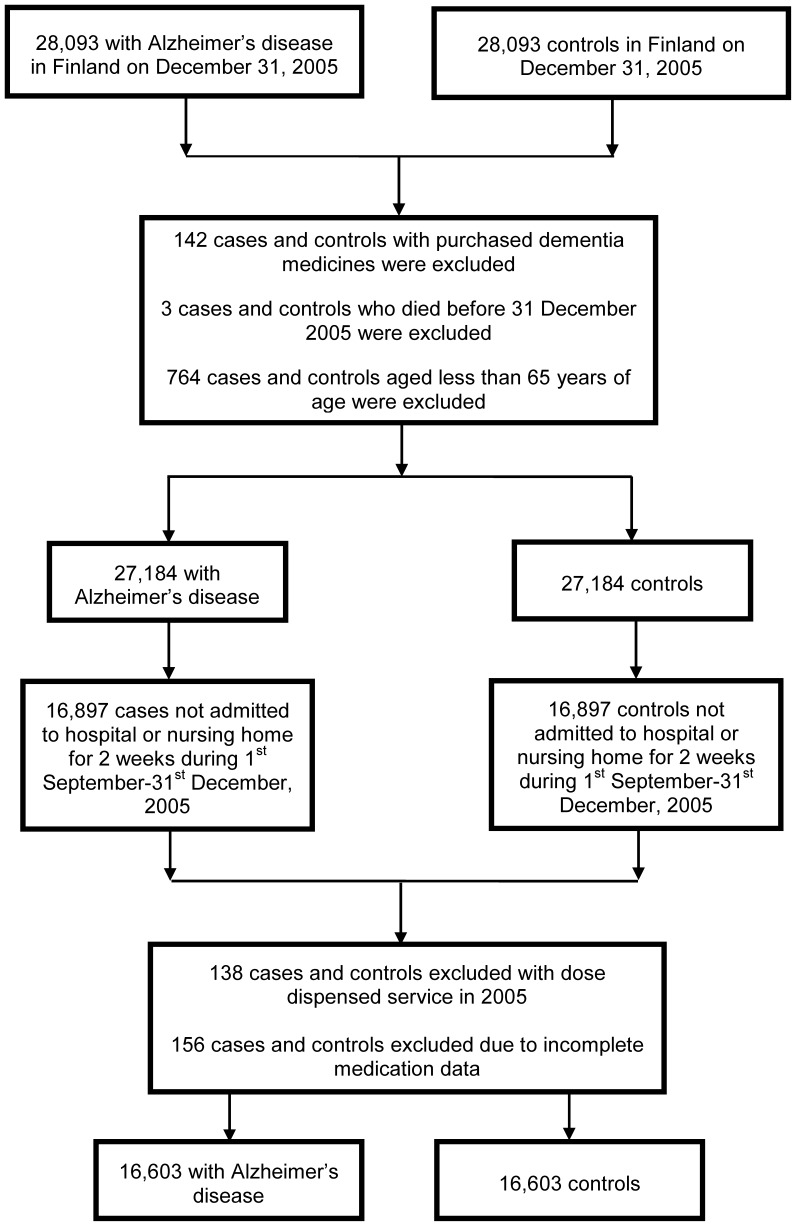
Flowchart of community-dwelling people with and without Alzheimer’s disease (AD) in Finland, and people included in the present analysis.

For each person with AD the SII also identified an individually matched comparison person. The matching was performed according to age (+/− one year), sex and region of residence. There were 912 matched pairs excluded from the analyses (764 pairs were aged less than 65 years, 142 comparison persons received an AD diagnosis in AD in 2006 which was backdated by the SII to 2005, and three persons died prior to 1 January 2006) (Figure 1). Moreover, for investigating the impact of drug exposure on hospitalization and death the dataset was further restricted to 16,897 matched pairs in which neither person was admitted to hospital or a nursing home for two weeks or more during the period 1 September-31 December 2005. This was because the Finnish National Prescription Register does not capture records of drugs dispensed to hospital inpatients. Pairs in which one person was hospitalized for a total of less than two weeks were included in the analyses because short term hospitalization or nursing home stay is commonly provided as a form of respite care for persons with AD in Finland. Moreover, another 294 matched pairs were excluded (138 matched pairs in which one person used dose dispensing services meaning data on dispensing volume were not available, and 156 matched pairs in which one person had otherwise incomplete dispensing data), giving a final sample size of 16, 603 matched pairs of people with and without AD.

### Drug Exposure

Records of all drugs purchased by the people with AD and their individually matched comparison person from 1 September-31 December 2005 were extracted from the National Prescription Register. Drugs were categorized according to the World Health Organization (WHO) Anatomical Therapeutic Chemical (ATC) classification system. The DBI, a measure of exposure to drugs with anticholinergic or sedative effects was calculated using the equation below;
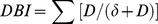
where D represents the daily dose and δ the minimum recommended daily dose reported by the Finnish registered drug product information. The daily dose was estimated from the total quantity dispensed, by multiplying the strength (mg) and quantity dispensed from 1^st^ September to 31^st^ December 2005, divided by the time period over which the drug was taken (four months). The rationale for this time frame was that all drugs would be dispensed at least once because Finnish regulations permit a maximum of three months’ supply of a drug to be dispensed in a single purchase. The ATC codes were used to screen for anticholinergic and sedative drugs.

### Comorbidities

Diagnostic data that were extracted from the Special Reimbursement Register included AD, cardiovascular disease (heart failure, hypertension, coronary artery disease, and arrhythmias), diabetes, respiratory disease (chronic asthma and chronic obstructive pulmonary disease), psychosis, musculoskeletal disorders (rheumatoid arthritis and other connective tissue disease) and cancer. Rather than adjusting for the presence of medical conditions, a comorbidity score was computed using the Charlson Comorbidity Index as a reference [26]. A modified comorbidity score was calculated using the following diseases: heart failure, coronary artery disease, diabetes I or II, chronic asthma or chronic obstructive pulmonary disease, disseminated connective tissue diseases, rheumatoid arthritis and other comparable conditions (score of one); uremia requiring dialysis, severe anemia in connection with chronic renal failure, leukemia and other malignant diseases of blood and bone marrow including malignant diseases of the lymphatic system, cancer including breast and prostate cancers, female genital tract cancer and malignant neoplasms (score of two).

### Study Outcomes

For this study, mortality and hospitalization data over one year, from 1^st^ January to 31^st^ December 2006 were obtained. Mortality data were extracted from the National Population Register for all people with a verified AD diagnosis on December 31^st^ 2005. Hospitalization data were extracted from the National Hospital Discharge Register. All hospital admissions were recorded and analyzed according to the aggregated length of hospital stay during 2006 and the number of separate admissions in 2006.

### Statistical Analysis

Characteristics of the study sample were summarized as means (range) or counts (percentage). In this sample, DBI was tested as a categorical ordinal variable [none (0), low (>0 to <1), and high (≥1)] and as a continuous variable. Differences in hospitalization and mortality with increasing DBI exposure were compared using χ^2^-tests. Univariate and multivariate Cox regressions analyses were conducted to determine the unadjusted and adjusted hazard ratios (HR) with 95% confidence intervals (CI) for the effects of DBI exposure on mortality. The assumptions of the final models were checked by using Akaike’s Information Criterion (AIC). The analysis was censored at the end of the 12-month follow-up period. Two different measures were used to investigate the association between DBI and hospitalization: (i) the aggregated length of hospital stay during 2006 and (ii) number of separate hospitalizations in 2006. As the assumptions for the Poisson regression models were not met, and negative binomial regression was inadequate because of excessive zeros, the zero-inflated negative binomial regression analysis was used to generate incidence rate ratios (IRRs), with 95% confidence intervals (CIs) for the association between DBI and length of hospital stay and number of hospital admissions. These analyses were performed for all different categories of DBI exposure. The analyses were censored for each participant at time of death or the end of the follow-up, whichever occurred first. Multivariate models were adjusted for age, gender and modified grouped comorbidity index. We repeated the analysis for people with AD to account for disease severity. The time since AD diagnosis was used as a surrogate measure of disease severity [27]. These analyses were performed to assess possible confounding by indication where anticholinergic and sedatives may be prescribed to manage symptoms in advanced AD and at the end-of-life (e.g. delirium, agitation). Finally, to assess the robustness of our analyses, we performed two sensitivity analyses. The first sensitivity analysis was performed including all participants aged 65 and over (n = 26,648). The second sensitivity analyses involved excluding all matched pairs in which either person was admitted to hospital or a nursing home during the period 1 September-31 December 2005 (n = 11,924). A two-sided p-value of <0.05 was used to indicate statistical significance in all analyses. Data were analyzed using SAS version 9.3 (SAS Institute Inc., Cary, North Carolina).

## Results

**Figure 2:**
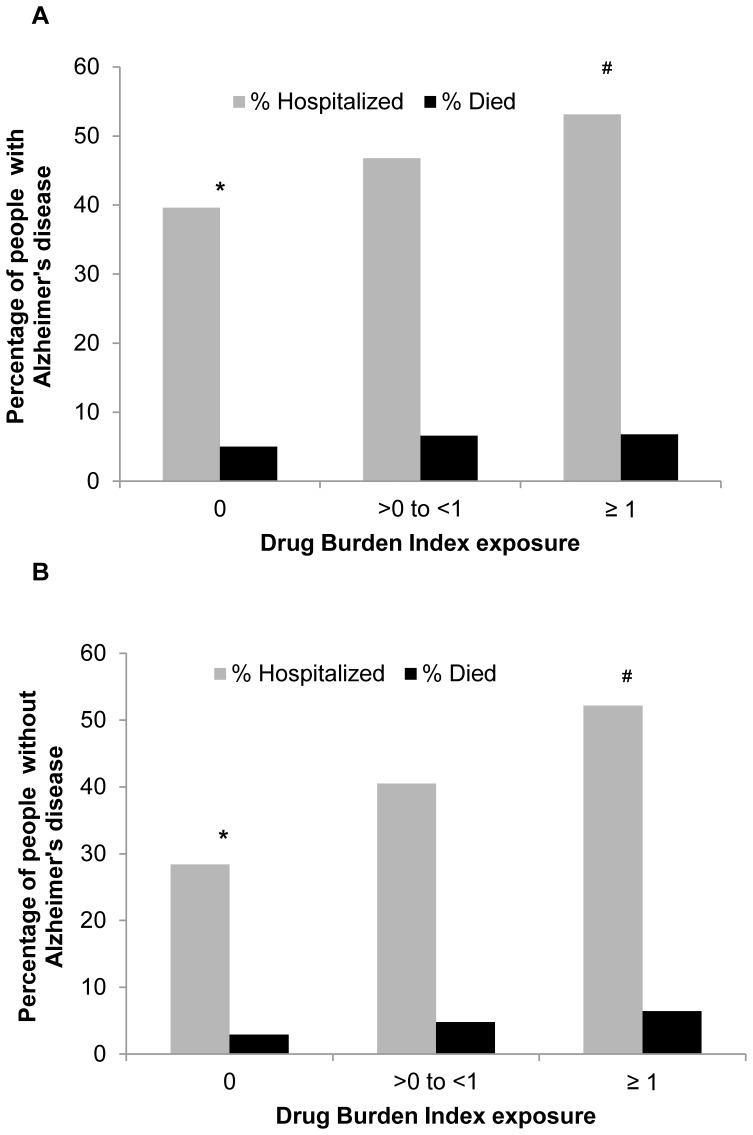
Percentage of people with and without Alzheimer’s disease hospitalized (2A) and died (2B) with increasing Drug Burden Index exposure. *Chi-square tests for differences between the groups: p<0.0001 for hospitalization and death. ^#^Trend test statistics between the groups: p<0.0001.

The characteristics of the study sample are described in Table 1. The mean age of people with and without AD was 79.2 years. There were more women (66.5%) than men among people with and without AD. The prevalence of the major diagnoses was similar in both people with and without AD. In relation to drug exposure, people with AD (51.4%) were more likely to be exposed to DBI drugs than people without AD (33.3%). A list of the most prevalent anticholinergic and sedative drugs in people with and without AD is provided in Table 1 (see Table S1). In relation to DBI groups, 16.0% of people with AD had a high DBI exposure (≥1) compared with 8.8% of people without AD. People with AD were more likely to be hospitalized (44.3%) and die (5.8%, equivalent to death rates of 6.0 per 100 persons-years) over one year, compared with people without AD (33.4% hospitalized; 3.7% died, equivalent to death rates of 3.7 per 100 persons-years). Increasing DBI exposure was associated with increased hospitalization and death rates in both people with and without AD (Figure 2).

**Table 1:** Baseline characteristics of the study sample.

Characteristic	People with Alzheimer’s disease (n = 16,603)	People without Alzheimer’s (n = 16,603)
Age, mean (range)	79.2 (65–101)	79.2 (65–101)
Age groups, n (%)		
65–74	3483 (21.0)	3483 (21.0)
75–84	10,120 (61.0)	10,120 (61.0)
≥85	3000 (18.1)	3000 (18.1)
Gender, n (%)		
Men	5566 (33.5)	5566 (33.5)
Women	11,037 (66.5)	11,037 (66.5)
Diagnoses, n (%)		
Cardiovascular disease*	8490 (51.1)	8920 (53.7)
Diabetes	2121 (12.8)	2114 (12.7)
Respiratory disease∧	1278 (7.7)	1604 (9.7)
Cancer	785 (4.7)	1065 (6.4)
Modified Comorbidity Index, mean (range)	0.7 (0–6)	0.7 (0–7)
Modified Comorbidity Index groups, n (%)		
0	9343 (56.3)	8725 (52.6)
1	4543 (27.4)	4705 (28.3)
≥2	2717 (16.4)	3173 (19.1)
Exposed to DBI drugs, n (%)		
Yes	8540 (51.4)	5529 (33.3)
No	8063 (48.6)	11,074 (66.7)
DBI score, mean (SD)	0.45 (0.59)	0.27 (0.49)
DBI groups, n (%)		
0	8063 (48.6)	11,074 (66.7)
>0 to <1	5878 (35.4)	4091 (24.6)
≥1	2662 (16.0)	1438 (8.7)
Died in 2006, n (%)		
Yes	971 (5.8)	607 (3.7)
No	15,632 (94.2)	15, 996 (96.3)
Hospitalized in 2006, n (%)		
Yes	7356 (44.3)	5547 (33.4)
No	9247 (55.7)	11,056 (66.6)
Length of hospital stay, mean (range)	16.1 (0–365)	7.1 (0–365)
Number of hospital admissions, mean (range)	0.8 (0–40)	0.6 (0–26)

DBI = drug burden index.

Cardiovascular disease includes heart failure, hypertension, coronary artery disease, and arrhythmias.

Respiratory disease includes chronic asthma and chronic obstructive pulmonary disease.

The results of the Cox models for the association of the DBI exposure with mortality are presented in Table 2. Among people with AD, for every unit increase in DBI (equivalent to exposure to two or more anticholinergic and sedative drugs at minimum effective dose), the adjusted HR for mortality was 1.21 (95%CI: 1.09–1.33). Among people without AD, for every unit increase in DBI, the adjusted HR for mortality was 1.37 (95%CI: 1.20–1.56). When using no DBI exposure as the reference group, adjusted HR for mortality for high DBI group (≥1) was 1.34 (95%CI: 1.13–1.60) in people with AD compared with an adjusted HR of 1.75 (95%CI: 1.39–2.22) in people without AD.

**Table 2:** The association of Drug Burden Index exposure with death over one year.

	People with Alzheimer’s disease (n = 16,603)		People without Alzheimer’s disease (n = 16,603)	
	Mortality outcome		Mortality outcome	
Cox regression models	Unadjusted HR (95%CI)	Adjusted HR (95%CI)^a^	Unadjusted HR (95%CI)	Adjusted HR (95%CI)
DBI, continuous score	1.22 (1.11–1.34)	1.21 (1.09–1.33)	1.53 (1.36–1.73)	1.37 (1.20–1.56)
DBI groups^b^				
0	1.00	1.00	1.00	1.00
>0 to <1	1.35 (1.17–1.55)	1.29 (1.12–1.48)	1.71 (1.43–2.04)	1.41 (1.18–1.68)
≥1	1.39 (1.16–1.65)	1.34 (1.13–1.60)	2.28 (1.81–2.88)	1.75 (1.39–2.22)

^a^ Adjusted for age, gender and modified grouped comorbidity score.

^b^ P-value for trend: p<0.0001.

Reference group = 1.

CI = confidence interval; DBI = drug burden index; HR = hazard ratios.

The findings of the zero-inflated negative binomial models for the association of DBI exposure with aggregated length of hospital stay and number of separate hospital admissions are shown in Table 3. For every unit increase in DBI, the adjusted IRR for length of hospital stay in people with AD was 1.06 (95%CI: 0.99–1.12). When using no DBI exposure as the reference group in people with AD, the adjusted IRR for high DBI group (≥1) was 1.15 (95%CI: 1.05–1.26). Among people without AD, for every unit increase in DBI the adjusted IRR for length of hospital stay was 1.35 (95%CI: 1.24–1.46). In people with AD, the number of separate hospital admissions increased with higher DBI exposure, with an adjusted IRR of 1.22 (95%CI: 1.17–1.27). Among people without AD, for every unit increase in DBI, the adjusted IRR for higher number of hospital admissions was 1.36 (95%CI: 1.28–1.43). There was a dose-response relationship between higher DBI exposure and higher number of separate hospital admissions in people with and without AD.

**Table 3:** Zero-inflated negative binomial regression for the association of Drug Burden Index exposure with length of hospital stay and number of hospital admissions over one year.

	People with Alzheimer’s disease (n = 16,603)		People without Alzheimer’s disease (n = 16,603)	
Length of hospital stay	Unadjusted IRR (95%CI)	Adjusted IRR (95%CI)^a^	Unadjusted IRR (95%CI)	Adjusted IRR (95%CI)
DBI, continuous score	1.05 (0.99–1.11)	1.06 (0.99–1.12)	1.28 (1.18–1.38)	1.35 (1.24–1.46)
DBI groups^b^				
0	1.00	1.00	1.00	1.00
>0 to <1	1.02 (0.95–1.10)	1.02 (0.95–1.10)	1.19 (1.07–1.31)	1.19 (1.07–1.32)
≥1	1.13 (1.03–1.24)	1.15 (1.05–1.26)	1.54 (1.34–1.76)	1.63 (1.41–1.88)
**Number of hospital admissions**	**Unadjusted IRR (95%CI)**	Adjusted IRR (95%CI)*	**Unadjusted IRR (95%CI)**	**Adjusted IRR (95%CI)**
DBI, continuous score	1.27 (1.21–1.33)	1.22 (1.17–1.27)	1.43 (1.34–1.52)	1.36 (1.28–1.43)
DBI groups^b^				
0	1.00	1.00	1.00	1.00
>0 to <1	1.25 (1.17, 1.35)	1.18 (1.11–1.26)	1.37 (1.26–1.49)	1.33 (1.24–1.43)
≥1	1.55 (1.43–1.69)	1.41 (1.31–1.52)	1.79 (1.61–2.00)	1.64 (1.49–1.80)

^a^ Adjusted for age, gender and modified grouped comorbidity score.

^b^ P-value for trend: p<0.0001.

Reference group = 1.

CI = confidence interval; DBI = drug burden index; IRR = incidence rate ratio.

A similar risk of death and hospitalization were observed among people with AD when analysis were adjusted for time since diagnosis. The results of the two sensitivity analyses also showed little change in the HRs estimates (*results available on request from the authors*). The relationship between DBI exposure and mortality remained the same in a subgroup excluding all matched pairs in which either person was admitted to hospital or a nursing home during the study period. However, in the total study population, higher DBI exposure was associated with increased mortality in people without AD only.

## Discussion

This is the first national population-based study to investigate the association between cumulative use of anticholinergic and sedative drugs with hospitalization and mortality in older people with AD. The main finding of this study was that there is dose-response relationship between higher DBI exposure and hospitalization and mortality in both people with and without AD. Increasing DBI exposure was associated with longer length of hospital stay and higher number of admissions in people with and without AD. These findings are of particular clinical importance given the high prevalence of anticholinergic and sedative drug use.

Previous studies have identified up to 30% of all unplanned hospital admissions among people aged 75 years and older may be drug-related [28]. A recent US study reported that warfarin, insulin, oral antiplatelets, and oral hypoglycemics were responsible for 67% of hospitalizations for recognized ADEs [29]. We adjusted our analyses using a modified comorbidity index that considered diagnoses of cardiovascular disease and diabetes. Data pertaining to cardiovascular disease and diabetes were extracted from the Special Reimbursement Register. Physicians include each patient’s diagnoses in the Special Reimbursement Register so that they are eligible to receive reimbursed drugs for these indications. This means that concomitant use of cardiovascular and antidiabetic drugs was unlikely to have explained the adjusted results in our study. Unlike ADEs associated with warfarin, insulin, oral antiplatelets, and oral hypoglycemics, ADEs associated with anticholinergics and sedatives (e.g. excess sedation, impaired balance, confusion) may go largely unrecognized by clinicians [30]. Our findings suggest that efforts to reduce hospitalizations related to ADEs should also include minimizing the use of anticholinergics and sedatives.

The findings of this national population study are consistent with some [9], [31], but not all [32], studies of anticholinergic use in more restricted small study samples. However, these smaller studies did not investigate the cumulative exposure to both anticholinergics and sedatives, and have not compared people with and without AD. The use of sedative drugs has been also associated with a greater risk of hospitalization and mortality in several studies [10], [29]. Higher DBI has been associated with impairments in objective measures of physical and cognitive function, and incident frailty [19], [20], [33], which in turn have been linked with increased risk of both hospitalization and mortality [34]. Exposure to DBI drugs has been also associated with increased hospitalization in community-dwelling older people [21], and longer length of hospital stay among older hospitalized patients [22]. Another study reported no association between increasing DBI exposure and mortality in older people living in residential aged care facilities in Australia [35]. There is evidence that use of multiple drugs and potentially inappropriate drugs, which includes drugs with anticholinergic and sedative properties, is associated with functional decline in older adults with dementia [2].

Even though the exposure to DBI drugs was higher in people with AD (51%) compared with people without AD (33%), the relative effects of DBI drugs on hospitalization and mortality were greater in people without AD. For every unit increase in DBI exposure, length of hospital stay increased by 6% and 35% among people with and without AD, respectively. These findings were also confirmed when we performed sensitivity analyses. It appears that, in people with AD the disease itself may drive the outcomes. This observation is supported by studies demonstrating the independent effects of AD on hospitalization [36] and mortality [37]. In a recent study conducted in the same Finnish population, AD was associated with an increased risk of mortality, independent of comorbidities [38].

In this study, it was not possible to analyze the prescribing indications for specific anticholinergic or sedative drugs because these data are not recorded in the Finnish National Prescription Register. Similarly, it was not possible to investigate specific causes of death from the data available to us. However, the most prevalent anticholinergic or sedative drugs in people with AD were antidepressants and sedative hypnotics. Similarly, in people without AD the most prevalent anticholinergic or sedative drugs were sedative hypnotics, opioid analgesics and antidepressants. These drug classes contribute to poorer functioning and quality of life in older people [39], [40]. The harms associated with specific drugs and the corresponding causes of death remains an important topic for future research.

Linking high-quality data from the Finnish National Prescription and Special Reimbursement Registers, and the Hospital Discharge Register provided a unique opportunity to study the impact of cumulative anticholinergic and sedative drugs in a real-world population of older people with and without AD. This is in contrast with randomized clinical trials, which commonly have particular inclusion and exclusion criteria, and exclude older people with comorbidities [39]. However, several important caveats need to be acknowledged when interpreting these findings, in particular the quantification of causality. As with any observational investigation, the possibility of residual confounding, confounding by disease severity and other unmeasurable factors cannot be excluded [39]. For instance, the observed associations may be due to underlying comorbidities, for which drugs were prescribed. Anticholinergic and sedative drugs may have been prescribed to manage symptoms in advanced AD and at the end of life (e.g. depression, delirium, agitation). We attempted to account for this within the AD cohort by adjusting the analysis for time since diagnosis. The contribution of other drug classes known to increase risks of hospitalization in older people was not investigated in this study [29], nor the concomitant use of anticholinergics and cholinesterase inhibitors, commonly identified in studies of older people [41]. It is unknown whether participants took DBI drugs at the time of hospitalization or death, which may have resulted in misclassification and bias of our results towards the null hypothesis. In addition, the modified comorbidity score was used to adjust for our analysis; hence we may have missed other common diseases which may result in increased hospitalization and mortality in older people. Moreover, data on AD severity were not available and some of the matched comparison people may have had undiagnosed AD or other types of dementias.

The analyses were conducted among community-dwelling people with and without AD in Finland. This means the results may not necessarily be generalizable to other countries or settings, where the utilization patterns of anticholinergic and sedative drugs may be different. The Finnish National Prescription Register only includes records of drugs dispensed to those in the community. Thus, to accurately assess drug use we excluded pairs of people with and without AD in which either person was hospitalized for two-weeks or more during the period over which drug exposure was assessed. Nevertheless, the demographic characteristics of people with AD included in the analyses were similar to whole population of people with AD in Finland.

The analyses were performed using data from health registers [23]. Health registers are not subject to recall bias, however, dispensed drugs may not have necessarily been taken by the study participants. This means that exposure to anticholinergic and sedative drugs may have been overestimated among both people with and without AD, although the prevalence of anticholinergic and sedative drug use in our study was similar to that reported in other settings [20], [42]. In relation to DBI calculations, the daily dose taken was an estimate calculated using the dispensed quantity; hence it may not reflect the true exposure. The DBI exposure may have been underestimated, as anticholinergic and sedative over the counter drugs are not captured in the Prescription Register.

In summary, in this large population based study, the prevalence of exposure to anticholinergic and sedative drugs was higher in older people with AD compared with people without AD. Moreover, these data imply a dose-response relationship of higher DBI exposure with hospitalization and mortality in both people with and without AD. While other studies are required to confirm these associations, our findings suggest that the use of anticholinergic and sedative drugs in older people with and without AD should be regularly reviewed. Future work is needed to determine whether reducing exposure to anticholinergic and sedative drugs is possible in people with and without AD, and, if possible, whether this will reduce hospitalization and mortality in people with and without AD.

## Supporting Information

Table S1 **The list of most common Drug Burden Index medications and indications in people with and without Alzheimer’s disease.**
(DOCX)

## References

[1] McLean AJ, Le Couteur DG (2004) Aging biology and geriatric clinical pharmacology. Pharmacol Rev 56: 163–184. 15169926 10.1124/pr.56.2.4

[2] Lau DT, Mercaldo ND, Shega JW, Rademaker A, Weintraub S (2011) Functional decline associated with polypharmacy and potentially inappropriate medications in community-dwelling older adults with dementia. Am J Alzheimers Dis Other Demen 26: 606–615. 22207646 10.1177/1533317511432734 PMC3298080

[3] Lu CJ, Tune LE (2003) Chronic exposure to anticholinergic medications adversely affects the course of Alzheimer disease. Am J Geriatr Psychiatry 11: 458–461. 12837675

[4] Onder G, Bonassi S, Abbatecola AM, Folino-Gallo P, Lapi F, et al.. (2013) High Prevalence of Poor Quality Drug Prescribing in Older Individuals: A Nationwide Report From the Italian Medicines Agency (AIFA). J Gerontol A Biol Sci Med Sci. Aug 2. [Epub ahead of print]. 10.1093/gerona/glt118 23913935

[5] Andersen F, Viitanen M, Halvorsen DS, Straume B, Engstad TA (2011) Co-morbidity and drug treatment in Alzheimer’s disease. A cross sectional study of participants in the dementia study in northern Norway. BMC Geriatr 11: 58. 21970467 10.1186/1471-2318-11-58 PMC3204237

[6] Tjia J, Rothman MR, Kiely DK, Shaffer ML, Holmes HM, et al (2010) Daily medication use in nursing home residents with advanced dementia. J Am Geriatr Soc 58: 880–888. 20406320 10.1111/j.1532-5415.2010.02819.x PMC2910133

[7] Lau DT, Mercaldo ND, Harris AT, Trittschuh E, Shega J, et al (2010) Polypharmacy and potentially inappropriate medication use among community-dwelling elders with dementia. Alzheimer Dis Assoc Disord 24: 56–63. 19561441 10.1097/WAD.0b013e31819d6ec9 PMC2837122

[8] Laitinen ML, Bell JS, Lavikainen P, Lonnroos E, Sulkava R, et al (2011) Nationwide study of antipsychotic use among community-dwelling persons with Alzheimer’s disease in Finland. Int Psychogeriatr 23: 1623–1631. 21867581 10.1017/S1041610211001621

[9] Panula J, Puustinen J, Jaatinen P, Vahlberg T, Aarnio P, et al (2009) Effects of potent anticholinergics, sedatives and antipsychotics on postoperative mortality in elderly patients with hip fracture: a retrospective, population-based study. Drugs Aging 26: 963–971. 19848441 10.2165/11317660-000000000-00000

[10] Kripke DF, Langer RD, Kline LE (2012) Hypnotics’ association with mortality or cancer: a matched cohort study. BMJ Open 2: e000850. 10.1136/bmjopen-2012-000850 PMC3293137 22371848

[11] Taipale HT, Bell JS, Gnjidic D, Sulkava R, Hartikainen S (2012) Sedative load among community-dwelling people aged 75 years or older: association with balance and mobility. J Clin Psychopharmacol 32: 218–224. 22367651 10.1097/JCP.0b013e3182485802

[12] Gnjidic D, Le Couteur DG, Hilmer SN, Cumming RG, Blyth FM, et al.. (2012) Sedative load and functional outcomes in community-dwelling older Australian men: the CHAMP study. Fundam Clin Pharmacol Jul 7. [Epub ahead of print]. 10.1111/j.1472-8206.2012.01063.x 22849300

[13] Landi F, Russo A, Liperoti R, Cesari M, Barillaro C, et al (2007) Anticholinergic drugs and physical function among frail elderly population. Clin Pharmacol Ther 81: 235–241. 17192773 10.1038/sj.clpt.6100035

[14] Gray SL, LaCroix AZ, Hanlon JT, Penninx BW, Blough DK, et al (2006) Benzodiazepine use and physical disability in community-dwelling older adults. J Am Geriatr Soc 54: 224–230. 16460372 10.1111/j.1532-5415.2005.00571.x PMC2365497

[15] Boudreau RM, Hanlon JT, Roumani YF, Studenski SA, Ruby CM, et al (2009) Central nervous system medication use and incident mobility limitation in community elders: the Health, Aging, and Body Composition study. Pharmacoepidemiol Drug Saf 18: 916–922. 19585466 10.1002/pds.1797 PMC2904745

[16] Hilmer SN, Gnjidic D (2013) Rethinking psychotropics in nursing homes. Med J Aust 198: 77. 23373484 10.5694/mja12.11593

[17] Hilmer SN, Mager DE, Simonsick EM, Cao Y, Ling SM, et al (2007) A drug burden index to define the functional burden of medications in older people. Arch Intern Med 167: 781–787. 17452540 10.1001/archinte.167.8.781

[18] Lieberman JA 3rd (2004) Managing anticholinergic side effects. J Clin Psychiatry 6: 20–23. PMC487008 16001097

[19] Gnjidic D, Hilmer SN, Blyth F, Naganathan V, Cumming RG, et al (2012) High risk prescribing and incidence of frailty among older community-dwelling men. Clin Pharmacol Ther 91: 521–528. 22297385 10.1038/clpt.2011.258

[20] Gnjidic D, Le Couteur DG, Abernethy DR, Hilmer SN (2012) Drug Burden Index and Beers criteria: Impact on functional outcomes in older people living in self-care retirement villages. J Clin Pharm: 258–265. 10.1177/0091270010395591 21292625

[21] Lonnroos E, Gnjidic D, Hilmer SN, Bell JS, Kautiainen H, et al (2012) Drug Burden Index and hospitalization among community-dwelling older people. Drugs Aging 29: 395–404. 22530705 10.2165/11631420-000000000-00000

[22] Lowry E, Woodman RJ, Soiza RL, Hilmer SN, Mangoni AA (2012) Drug burden index, physical function, and adverse outcomes in older hospitalized patients. J Clin Pharmacol 52: 1584–1591. 22167569 10.1177/0091270011421489

[23] Furu K, Wettermark B, Andersen M, Martikainen JE, Almarsdottir AB, et al (2010) The Nordic countries as a cohort for pharmacoepidemiological research. Basic Clin Pharmacol Toxicol 106: 86–94. 19961477 10.1111/j.1742-7843.2009.00494.x

[24] Sund R (2012) Quality of the Finnish Hospital Discharge Register: A systematic review. Scand J Public Health 40: 505–515. 22899561 10.1177/1403494812456637

[25] McKhann G, Drachman D, Folstein M, Katzman R, Price D, et al (1984) Clinical diagnosis of Alzheimer’s disease: report of the NINCDS-ADRDA Work Group under the auspices of Department of Health and Human Services Task Force on Alzheimer’s Disease. Neurology 34: 939–944. 6610841 10.1212/wnl.34.7.939

[26] Charlson ME, Pompei P, Ales KL, MacKenzie CR (1987) A new method of classifying prognostic comorbidity in longitudinal studies: development and validation. J Chronic Dis 40: 373–383. 3558716 10.1016/0021-9681(87)90171-8

[27] Jost BC, Grossberg GT (1995) The natural history of Alzheimer’s disease: a brain bank study. J Am Geriatr Soc 43: 1248–1255. 7594159 10.1111/j.1532-5415.1995.tb07401.x

[28] Runciman WB, Roughead EE, Semple SJ, Adams RJ (2003) Adverse drug events and medication errors in Australia. Int J Qual Health Care 15 Suppl 1i49–59. 14660523 10.1093/intqhc/mzg085

[29] Budnitz DS, Lovegrove MC, Shehab N, Richards CL (2011) Emergency hospitalizations for adverse drug events in older americans. N Engl J Med 365: 2002–2012. 22111719 10.1056/NEJMsa1103053

[30] Gnjidic D, Hilmer SN (2012) Emergency hospitalizations for adverse drug events. N Engl J Med 366: 859; author reply 859–860. 10.1056/NEJMc1114768 22375985

[31] Mangoni AA, van Munster BC, Woodman RJ, de Rooij SE (2013) Measures of anticholinergic drug exposure, serum anticholinergic activity, and all-cause postdischarge mortality in older hospitalized patients with hip fractures. Am J Geriatr Psychiatry: 785–793. 10.1016/j.jagp.2013.01.012 23567395

[32] Kumpula EK, Bell JS, Soini H, Pitkala KH (2011) Anticholinergic drug use and mortality among residents of long-term care facilities: a prospective cohort study. J Clin Pharmacol 51: 256–263. 20489026 10.1177/0091270010368410

[33] Hilmer SN, Mager DE, Simonsick EM, Ling SM, Windham BG, et al.. (2009) Drug burden index score and functional decline in older people. Am J Med 122: 1142–1149 e1142. 10.1016/j.amjmed.2009.02.021 PMC3263511 19958893

[34] Studenski S, Perera S, Patel K, Rosano C, Faulkner K, et al (2011) Gait speed and survival in older adults. JAMA 305: 50–58. 21205966 10.1001/jama.2010.1923 PMC3080184

[35] Wilson NM, Hilmer SN, March LM, Chen JS, Gnjidic D, et al (2012) Associations between Drug Burden Index and Mortality in Older People in Residential Aged Care Facilities. Drugs Aging 29: 157–165. 22276959 10.2165/11598570-000000000-00000

[36] Zilkens RR, Spilsbury K, Bruce DG, Semmens JB (2009) Linkage of hospital and death records increased identification of dementia cases and death rate estimates. Neuroepidemiology 32: 61–69. 19001798 10.1159/000170908

[37] Wolfson C, Wolfson DB, Asgharian M, M’Lan CE, Ostbye T, et al (2001) A reevaluation of the duration of survival after the onset of dementia. N Engl J Med 344: 1111–1116. 11297701 10.1056/NEJM200104123441501

[38] Lonnroos E, Kyyronen P, Bell JS, van der Cammen TJ, Hartikainen S (2013) Risk of death among persons with Alzheimer’s disease: a national register-based nested case-control study. J Alzheimers Dis 33: 157–164. 22914589 10.3233/JAD-2012-120808

[39] Hilmer SN, Gnjidic D, Abernethy DR (2012) Pharmacoepidemiology in the postmarketing assessment of the safety and efficacy of drugs in older adults. J Gerontol A Biol Sci Med Sci 67: 181–188. 21653991 10.1093/gerona/glr066

[40] Hilmer SN, Gnjidic D (2009) The effects of polypharmacy in older adults. Clin Pharmacol Ther 85: 86–88. 19037203 10.1038/clpt.2008.224

[41] Johnell K, Fastbom J (2008) Concurrent use of anticholinergic drugs and cholinesterase inhibitors: register-based study of over 700,000 elderly patients. Drugs Aging 25: 871–877. 18808211 10.2165/00002512-200825100-00006

[42] Gnjidic D, Bell JS, Hilmer SN, Lonnroos E, Sulkava R, et al (2012) Drug Burden Index associated with function in community dwelling older people living in Finland: A cross-sectional study. Ann Med 44: 458–467. 21495785 10.3109/07853890.2011.573499

