# Supplementary material for: Impact of High Risk Drug Use on Hospitalization and Mortality in Older People with and without Alzheimer’s Disease: A National Population Cohort Study
Source: PLoS One. 2014 Jan 13;9(1):e83224. doi: 10.1371/journal.pone.0083224 (PMC3890276; doi:10.1371/journal.pone.0083224)
Supplement: Table S1 — The list of most common Drug Burden Index medications and indications in people with and without Alzheimer’s disease. (DOCX) [file pone.0083224.s001.docx]

**Table S1. The list of most common Drug Burden Index medications and indications in people with and without Alzheimer’s disease.**

| **People with Alzheimer’s disease** | | | **People without Alzheimer’s disease** | | |
| --- | --- | --- | --- | --- | --- |
| **Medication** | **Prevalence (%)*** | **Common indication** | **Medication** | **Prevalence (%)*** | **Common indication** |
| Citalopram (anticholinergic) | 3.07% | Depression | Zopiclone (sedative) | 2.23% | Insomnia |
| Zopiclone (sedative) | 2.47% | Insomnia | Temazepam (sedative) | 1.25% | Insomnia |
| Mirtazapine (anticholinergic) | 1.71% | Depression | Tamsulosin (anticholinergic) | 1.07% | Management of LUTS |
| Temazepam (sedative) | 1.66% | Insomnia | Tramadol (sedative) | 0.60% | Pain |
| Quetiapine (anticholinergic) | 1.57% | Schizophrenia; Bipolar disorders | Oxazepam (sedative) | 0.56% | Anxiety |
| Risperidone (sedative) | 1.54% | Schizophrenia; Related psychosis; behavioural disturbance in dementia | Mirtazapine (anticholinergic) | 0.43% | Depression |
| Tamsulosin (anticholinergic) | 1.48% | Management of LUTS | Tolterodine (anticholinergic) | 0.42% | Overactive bladder |
| Escitalopram (anticholinergic) | 1.01% | Depression | Tiotropium (anticholinergic) | 0.34% | COPD |
| Oxazepam (sedative) | 0.93% | Anxiety | Alfuzosin (anticholinergic) | 0.25% | BPH |
| Tolterodine (anticholinergic) | 0.71% | Overactive bladder | Levodopa combinations (sedative) | 0.24% | Parkinson’s disease |
| Levodopa combinations (sedative) | 0.58% | Parkinson’s disease | Cetirizine (anticholinergic) | 0.24% | Allergic rhinitis |
| Lorazepam (sedative) | 0.49% | Anxiety | Zolpidem (sedative) | 0.24% | Insomnia |
| Alfuzosin (anticholinergic) | 0.36% | BPH | Alprazolam (sedative) | 0.23% | Anxiety |
| Sertraline (sedative) | 0.35% | Depression | Betahistine (anticholinergic) | 0.21% | Meniere's syndrome |
| Zolpidem (sedative) | 0.27% | Insomnia | Pregabalin (sedative) | 0.20% | Seizures, Neuropathic pain |

*percentage of total number of dispensed medicines.

COPD, chronic obstructive pulmonary disorder.

LUTS, lower urinary tract symptoms.

BPH, Benign prostatic hyperplasia.

A
